# Lipoprotein-Associated Phospholipase A2: A Novel Contributor in Sjögren’s Syndrome-Related Lymphoma?

**DOI:** 10.3389/fimmu.2021.683623

**Published:** 2021-06-18

**Authors:** Adrianos Nezos, Charalampos Skarlis, Anna Psarrou, Konstantinos Markakis, Panagiotis Garantziotis, Asimina Papanikolaou, Fotini Gravani, Michael Voulgarelis, Athanasios G. Tzioufas, Michael Koutsilieris, Haralampos M. Moutsopoulos, Eleni Kotsifaki, Clio P. Mavragani

**Affiliations:** ^1^ Department of Physiology, School of Medicine, National and Kapodistrian University of Athens, Athens, Greece; ^2^ Division of Immunology and Rheumatology, Hannover Medical University, Hannover, Germany; ^3^ Department of Hemopathology, Evangelismos Hospital, Athens, Greece; ^4^ Department of Rheumatology, General Hospital of Athens “G.Gennimatas”, Athens, Greece; ^5^ Department of Pathophysiology, School of Medicine, National and Kapodistrian University of Athens, Athens, Greece; ^6^ Joint Academic Rheumatology Program, National and Kapodistrian University of Athens School of Medicine, Athens, Greece; ^7^ Chair Medical Sciences/Immunology, Academy of Athens, Athens, Greece

**Keywords:** Sjögren’s syndrome, lymphomagenesis, lipoprotein-associated phospholipase A2 (Lp-PLA2), serum biomarker, novel therapeutic target

## Abstract

**Background:**

B-cell non-Hodgkin’s lymphoma (B-NHL) is one of the major complications of primary Sjögren’s syndrome (SS). Chronic inflammation and macrophages in SS minor salivary glands have been previously suggested as significant predictors for lymphoma development among SS patients. Lipoprotein-associated phospholipase A2 (Lp-PLA2)—a product mainly of tissue macrophages—is found in the circulation associated with lipoproteins and has been previously involved in cardiovascular, autoimmune, and malignant diseases, including lymphoma.

**Objective:**

The purpose of the current study was to investigate the contributory role of Lp-PLA2 in B-NHL development in the setting of primary SS.

**Methods:**

Lp-PLA2 activity in serum samples collected from 50 primary SS patients with no lymphoma (SS-nL), 9 primary SS patients with lymphoma (SS-L), and 42 healthy controls (HC) was determined by detection of [^3^H]PAF degradation products by liquid scintillation counter. Moreover, additional sera from 50 SS-nL, 28 SS-L, and 32 HC were tested for Lp-PLA2 activity using a commercially available ELISA kit. Lp-PLA2 mRNA, and protein expression in minor salivary gland (MSG) tissue samples derived from SS-nL, SS-L patients, and sicca controls (SC) were analyzed by real-time PCR, Western blot, and immunohistochemistry.

**Results:**

Serum Lp-PLA2 activity was significantly increased in SS-L compared to both SS-nL and HC by two independent methods implemented [mean ± SD (nmol/min/ml): 62.0 ± 13.4 *vs* 47.6 ± 14.4 *vs* 50.7 ± 16.6, p-values: 0.003 and 0.04, respectively, and 19.4 ± 4.5 *vs* 15.2 ± 3.3 *vs* 14.5 ± 3.0, p-values: <0.0001, in both comparisons]. ROC analysis revealed that the serum Lp-PLA2 activity measured either by radioimmunoassay or ELISA has the potential to distinguish between SS-L and SS-nL patients (area under the curve [AUC]: 0.8022, CI [95%]: 0.64–0.96, p-value: 0.004 for radioimmunoassay, and AUC: 0.7696, CI [95%]: 0.66–0.88, p-value: <0.0001, for ELISA). Lp-PLA2 expression in MSG tissues was also increased in SS-L compared to SS-nL and SC at both mRNA and protein level. ROC analysis revealed that both MSG mRNA and protein Lp-PLA2 have the potential to distinguish between SS-nL and SS-L patients (area under the curve [AUC] values of 0.8490, CI [95%]: 0.71–0.99, p-value: 0.0019 and 0.9444, CI [95%]: 0.79–1.00, p- value: 0.0389 respectively). No significant difference in either serum Lp-PLA2 activity or MSG tissue expression was observed between SS-nL and HC.

**Conclusions:**

Lp-PLA2 serum activity and MSG tissue mRNA/protein expression could be a new biomarker and possibly a novel therapeutic target for B-cell lymphoproliferation in the setting of SS.

## Introduction

Sjögren’s syndrome (SS) is a chronic autoimmune disorder primarily affecting perimenopausal women. Dryness of the mouth and eyes and periductal lymphocytic cell infiltrates in the exocrine glands are the chief clinical and histopathological features. More than a half of SS patients experience systemic disease manifestations (1–3) and nearly 5% to 10% develop B-cell non-Hodgkin lymphoma resulting in increased mortality rates (4–6).

Deregulated inflammatory responses have been associated with lymphoma development in the setting of SS (7). Excessive lymphocytic infiltrates (8), heightened rates of IL-18–expressing cells (9), and tissue macrophages (10), elevated transcript levels of both interferon-γ (IFNγ) (11–13), and inflammasome molecules (14) in minor salivary gland (MSG) tissues, as well high serum levels of IL-18 and apoptosis-associated speck-like protein (ASC) (14, 15), have been all designated as key findings in SS patients at high risk for lymphoma development. Toward the same direction, several gene variants implicated in central inflammatory and IFN/B cell activation pathways, such as tumor necrosis factor alpha-induced protein 3 (*TNFAIP3*) variant (16, 17), three prime repair exonuclease 1 *(TREX-1)* (18), B-cell activating factor *(BAFF)* (19), and *BAFF-R* (20) have been shown to increase susceptibility for SS-related lymphoma.

A major inflammatory molecule, mainly produced during the process of monocyte differentiation into macrophages and involved in chronic inflammatory disorders including cardiovascular (21, 22), autoimmune/inflammatory (23, 24), and neoplastic (25) diseases, is lipoprotein-associated phospholipase A2 (Lp-PLA2), formerly called platelet-activating factor acetylhydrolase (PAF-AH). Lp-PLA2 belongs to the superfamily of esterase enzymes which mediate the production of free fatty acids and lysophospholipids from glycerophospholipids (26). Lp-PLA2 specifically functions as a catalyst in the hydrolysis of PAF, a plasma phospholipid with powerful inflammatory actions (27). Decreasing of biologically active phospholipids, such as PAF and oxidatively fragmented phospholipids (OxPL), or increasing oxidized and/or short/medium chain fatty acids and lyso-phosphatidylcholine (lyso-PC), have been postulated as key mechanisms through which Lp-PLA2 regulate signaling inflammatory events (28).

In the current study, we investigated the potential role of serum Lp-PLA2 activity, as well as MSG tissue mRNA and protein expression as a biomarker for B-NHL in the setting of primary SS.

## Patients and Methods

### Patients

In the present case-control study, serum samples derived from 100 primary SS patients with no history of lymphoma (SS-nL), 37 primary SS patients with a history of lymphoma (SS-L), and 74 healthy controls (HC) were included. Labial MSG biopsy tissues performed as part of the routine diagnostic evaluation of SS were available in 16 SS-nL, 12 SS-L, and 6 sicca controls (SC) (Department of Pathophysiology, School of Medicine, National and Kapodistrian University of Athens). All patients fulfilled the 2016 ACR/EULAR SS classification criteria (29) and were followed up in the Outpatient Rheumatology Clinics of the Department of Pathophysiology, Laiko General Hospital and the General Hospital of Athens G.Gennimatas, as well as by Prof HM Moutsopoulos. SC are individuals presenting with dryness of oral and/ocular mucosae without fulfilling classification criteria for SS. Based on the number of adverse predictors present at the time of diagnosis [salivary gland enlargement (SGE), lymphadenopathy, Raynaud’s phenomenon, anti-Ro/SSA or/and anti-La/SSB autoantibodies, rheumatoid factor (RF) positivity, monoclonal gammopathy, and C4 hypocomplementemia], SS patients were further classified into low risk (two or less adverse predictors), medium risk (three to six adverse predictors), and high risk for lymphoma development (seven adverse predictors) as previously described (30). SS-L patients were followed up in the Outpatient Hematology Clinic of the Department of Pathophysiology, School of Medicine, National and Kapodistrian University of Athens. Lymphoma diagnosis was based on World Health Organization classification criteria. Of the total 37 SS-L cases included in the study, 33 were of mucosa-associated lymphoid tissue type and four of diffuse large cell lymphomas (one in the first set and three in the second sample set). 15 SS-L patients had previously received either rituximab alone or combination with chemotherapy. Serum samples were collected, stored, and processed in the Department of Physiology, School of Medicine, National and Kapodistrian University of Athens. The study was approved by Laiko General Hospital of Athens and General Hospital of Athens Ethics Committee. All subjects gave informed consent in accordance with the Declaration of Helsinki.

Clinical, serological, and histopathological characteristics were recorded after thorough chart review as previously described (11). In the first SS set, data for the presence of plaque formation, measurements for intima media thickness (IMT), as a marker of subclinical atherosclerosis and classic risk factors for atherosclerosis were also available (31). The presence of subclinical atherosclerosis was defined by the presence of plaque and/or arterial wall thickening (defined as IMT score >0.90 mm in carotid and femoral arteries) as determined by ultrasound (iU22, Philips, Royal Philips Electronics of the Netherlands) (31).

### Methods

#### Measurement of Lp-PLA2 Activity

Lp-PLA2 activity was measured by liquid scintillation in human serum samples derived from 50 primary SS patients with no lymphoma (SS-nL), 9 primary SS patients with lymphoma (SS-L), and 42 healthy controls (HC) stored at −80°C, in the setting of a previous study exploring mechanisms of subclinical atherosclerosis in primary SS (31). The assay measured [^3^H]PAF degradation products in liquid scintillation counter. Briefly, each sample was incubated with 10 μM [^3^H]PAF in PBS, pH 7.4, for 10 min at 37°C. The lipids were extracted at the end of incubation by the method of Folch et al. (32), and the amount of radioactivity recovered in the aqueous phase was determined by liquid scintillation as previously described (33).

Moreover, additional sera from 50 SS-nL, 28 SS-L, and 32 HC were tested for Lp-PLA2 activity using a commercially available ELISA kit (PAF Acetylhydrolase Assay Kit, Cayman Chemical, USA), according to manufacturer’s instructions. All measurements were performed in duplicate at 412 nm using a plate reader (Versamax, Molecular Devices, USA). In this case, SS patients either with a history of lymphoma or not who were followed in the last 2 years in Hematology Unit, Department of Pathophysiology and the Outpatient Clinic, Department of Pathophysiology, respectively, were contacted, and blood was drawn for the study purposes after informed consent was obtained. Patients who had received chemotherapy or rituximab in the previous year were excluded from the study. The mean duration between sampling and lymphoma diagnosis was 6.1 ± 4.1 years.

#### MSG Tissue RNA and Protein Extraction

Total RNA was extracted from MSG tissue biopsies (stored at −80°C) by TRIzol Reagent (Thermo Scientific, USA) according to manufacturer’s instructions. The quantity and quality of RNA samples was spectrophotometrically recorded (Biospec Nano, Japan). A commercial preparation of total RNA from normal salivary glands pooled from 24 male/female Caucasians (ages 15–60 years; cause of death: sudden death) was used as a source of healthy RNA (Clontech Laboratories Inc, USA). Protein extraction from MSG tissues was performed by lysis of the tissue in radioimmunoprecipitation assay extraction buffer (RIPA buffer) supplemented with protease and phosphatase cocktail inhibitor and 1 mM PMSF (all reagents purchased from New England Biolabs, Canada). Pierce BCA Protein Assay Kit (Thermo Fisher Scientific, USA) was performed for determination of protein concentration of each sample.

#### cDNA Synthesis and Real-Time PCR

Total RNA obtained from MSG samples was reverse-transcribed using Superscript III reverse transcriptase system from Invitrogen (Thermo Fisher, USA). Complementary DNA samples were diluted 1:10 with nuclease free water (Qiagen, Germany) immediately after synthesis and stored at −20°C.

Quantitative Real-Time Polymerase Chain Reaction (qRT-PCR) was used to quantify specific cDNAs using the Bio-Rad IQ5 thermocycler and the Kapa Biosystems SYBR Green qPCR Master Mix kit (Kapa Biosystems, South Africa). Specific primers to amplify only cDNA (exon-intron spanning) for each gene were designed using the Beacon Designer software. Lp-PLA2 is encoded by the PLA2G7 gene. The sequences of each primer set are as follows: PLA2G7 forward primer: 5′-CTGCTATTGGCATTGACCTGGC-3′ and reverse primer 5′-AGGTAGAGCCAAGACTTGTCCC-3. The normalization gene GAPDH primers were the following: forward primer 5′-CATCACTGCCACCCAGAAGA-3; and reverse primer 5′-TCCACCACCCTGTTGCTGTA-3′. The reaction was carried out in a total volume of 20 μl per reaction and constituted of 2 μl of template cDNA, 0.4 μM of each primer, 10 μl 2× KAPA SYBR Green Mix (Kapa Biosystems, South Africa), and ultra-pure water. A two-step amplification protocol was applied starting with step 1 with one cycle at 95°C for 4 min followed by step 2 with 40 cycles at 95°C for 5 s and 63°C for 30 s. The specificity of the amplified products was determined by melting curve analysis. The threshold cycles (Ct) generated by the qPCR system were used to calculate relative gene expression levels between different samples. Briefly, the Ct of the target gene (or gene of interest) was subtracted from the Ct of the reference gene for the two groups and the relative expression of each sample was determined using the 2^−^ *^ΔΔCt^* method, as previously described (11). All reactions were performed in duplicate. MSG healthy RNA pool was the reference sample for MSG tissues.

#### Western Blot

An equal amount of total protein lysates (25 μg) was heated at 95°C for 5 min, electrophoresed on 10% sodium dodecyl sulfate (SDS)–polyacrylamide gel (29:1 acrylamide, Applichem, Germany) under denaturing conditions for 2 h, and transferred overnight at 4°C onto PVDF membranes (Bio-Rad, USA). The blots were treated with TBST blocking buffer (20 mmol/L Tris HCl, pH 7.6, 137 mmol/L NaCl, and 0.1% Tween 20) containing 5% nonfat dried milk at room temperature for 90 min and incubated overnight at 4°C with anti–Lp-PLA2 rabbit polyclonal Antibody primary antibody (Origene, USA) at 1:1.000 dilution in TBST containing 5% milk followed by GAPDH anti-mouse monoclonal antibody, as a reference protein expression (Santa Cruz Biotechnology). The blots were washed thrice for 5 min each time, followed by incubation with anti-rabbit IgG secondary goat antibody (Santa Cruz Biotechnology) or anti-mouse IgG secondary goat antibody conjugated to horseradish peroxidase at 1:2.000 dilution (Santa Cruz Biotechnology).

The blots were exposed on X-ray film after incubation with freshly made enhanced chemiluminescent substrate for 2 min (GAPDH) or 20 min (PLA2G7) (SuperSignal; Pierce/Thermo Scientific, USA). Densitometry analysis was performed using ImageJ image processing software (http://rsb.info.nih.gov/ij). As a positive control for the Lp-PLA2 protein expression was used a total protein lysate from human placenta tissue sample available in our laboratory (high RNA expression based on Human Protein Atlas, https://www.proteinatlas.org/ENSG00000146070-PLA2G7/tissue).

#### Immunohistochemistry

Thirteen formalin-fixed paraffin-embedded MSG tissue sections (5 μm) derived from four SS patients, 5 SS-L patients and four patients complaining of sicca symptoms without fulfilling histopathological diagnosis for SS served as SC were also included. A lymph node biopsy derived from a non-SS patient with marginal zone lymphoma was also included.

Immunohistochemical detection of Lp-PLA2 (PLA2G7) and CD68 was performed by a standard immunoperoxidase technique using the SignalStain Boost IHC Detection Reagent (HRP, Rabbit) (Cell Signaling, USA). Briefly, paraffin sections were rehydrated in successive baths of xylene, 100%, 96%, 80%, 70% ethanol, and distilled water and washed thrice with PBS (phosphate-buffered saline). Antigen retrieval was performed by microwaving for 10 min in 0.01 M citrate buffer (pH 6.0). Incubation for 10 min at room temperature with Power block Universal Blocking Reagent (BioGenex, USA) and 10 min with 3% H_2_O_2_ (BioGenex, USA) were performed to block non-specific antibody binding and endogenous peroxidase activity, respectively. Incubation of serial sections with Lp-PLA2 (PLA2G7 gene) rabbit polyclonal antibody (Origene, USA) at 1:25 dilution, CD68/PGM1 (DAKO, USA) mouse monoclonal antibody at 1:30 dilution and concentration-matched isotype control antibody (PharMingen, San Diego, CA) was performed for 30 min at room temperature. Polymer–horseradish peroxidise (HRP) Reagent (Cell Signaling, USA) was applied for 30 min at room temperature and after a washing step the substrate diaminobenzidine (DAB) solution (Cell Signalling, USA) was performed for 5 to 10 min. Biopsy sections were counterstained with hematoxylin for 2 min (Mayers Haematoxylin solution, Sigma Aldrich Inc, USA), dehydrated in successive baths of water, 70%, 80%, 96%, 100% ethanol, and xylene and coverslip mounted with two drops of Aqueous Mounting Media (Chembiotin, Canada). Negative control staining was performed by replacing primary antibody with PBS. Positive immunoreactivity appears as brown color. The intensity of immunoreactivity for Lp-PLA2 was determined as follows: 0 for no staining, 1 for weak intensity staining, 2 for intermediate intensity staining, 3 for strong intensity staining, as previously suggested (34).

As a positive control a human tonsil section sample was used, according to Lp-PLA2 (PLA2G7 gene) rabbit polyclonal antibody (Origene, USA) datasheet (data not shown).

#### Statistics

Two-group comparisons of continuous data were assessed using t-tests, or the Mann-Whitney test, when the data did not have a normal distribution. For the comparison of numerical variables between three groups, analysis of variance (ANOVA) test was implemented. For the comparison of categorical variables, Fisher’s exact test was implemented. Receiver operating characteristic (ROC) curve and corresponding area under the curve (AUC) were calculated. Difference was considered statistically significant if p<0.05. Statistical analysis was performed by SPSS v.25 package and Graph Pad Prism 8.0.

## Results

### Clinical and Serological Characteristics of Study Participants

Clinical, hematological, serological, and histopathological characteristics of SS-nL and SS-L patients included in the study are displayed in **Supplementary Tables 1, 2**. Demographics from healthy controls are also presented in **Supplementary Tables 1**, **2**. **Supplementary Table 3** summarizes the clinical characteristics of SS patients in radioimmunoassay and ELISA cohorts.

### Serum Lp-PLA2 Activity by Liquid Scintillation

**Figure 1:**
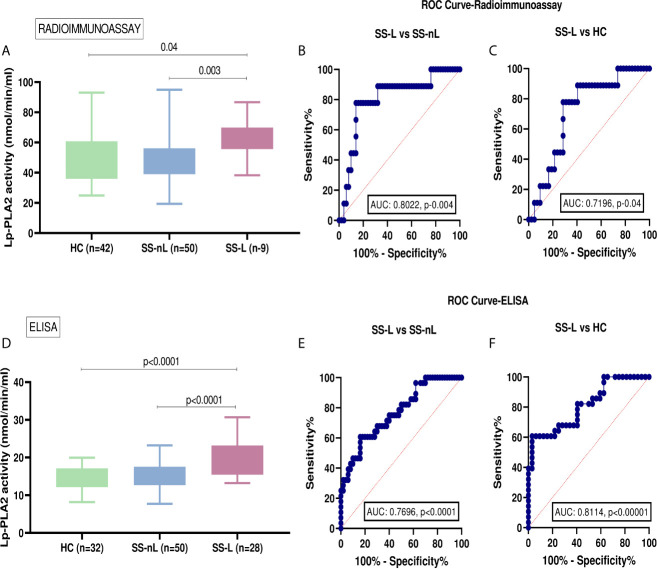
Serum Lp-PLA2 activity in the two SS sets of samples and receiver-operating characteristic analysis (ROC) curve analysis for both radioimmunoassay and ELISA protocols. **(A)** Increased Lp-PLA2 activity measured by liquid scintillation in SS-L patients compared to SS-nL (p-value: 0.003) and HC (p=0.04). **(B)** ROC analysis revealed that serum Lp-PLA2 activity measured by radioimmunoassay has the potential to distinguish between SS-L and SS-nL patients [area under the curve (AUC) = 0.8022, CI (95%): 0.64–0.96, p-value: 0.004]. **(C)** When ROC curves for the predictive models were fitted, AUC were 0.7196, CI (95%): 0.56–0.88, p-value: 0.04 for SS-L vs HC. **(D)** Increased Lp-PLA2 activity measured by ELISA in patients with SS-L compared to SS-nL patients (p-value: <0.0001) and HC (p-value: <0.0001). **(E)** ROC analysis showed that serum Lp-PLA2 activity measured by ELISA has the potential to distinguish between SS-L and SS-nL patients with AUC 0.7696, CI (95%): 0.66–0.88, p-value <0.0001 and **(F)** For SS-L patients vs HC, ROC curves showed an AUC 0.8114, CI (95%): 0.70–0.92, p-value < 0.0001. HC, healthy controls; SS-nL, Sjogren’s syndrome without lymphoma; SS-L, Sjogren’s syndrome complicated by lymphoma; ROC, receiver operating characteristic; AUC, area under the curve.

Serum Lp-PLA2 activity measured by liquid scintillation was found to be significantly increased in patients with SS-L compared to SS-nL group (**Figure 1A**), [mean ± SD (nmol/min/ml): 62.0 ± 13.4 *vs* 47.6 ± 14.4, p-value: 0.003], and HC [mean ± SD (nmol/min/ml): 62.0 ± 13.4 *vs* 50.7 ± 16.6, p-value: 0.04]. No statistically significant difference in Lp-PLA2 activity was observed between SS-nL patients and HC. Following comparison of the three groups by ANOVA testing, the resulting p-value was 0.036. ROC analysis showed that the serum Lp-PLA2 activity has the potential to distinguish between SS-L and SS-nL patients (area under the curve [AUC] = 0.8022, CI (95%): 0.64–0.96, p-value: 0.004 (**Figure 1B**). The corresponding value between SS-L and HC was 0.7196, CI (95%): 0.56–0.88, p-value: 0.04 (**Figure 1C**). As shown in **Supplementary Figure 1A**, no significant differences between low and medium risk were detected. No patient included in the present study belonged to the high risk for lymphoma development group. Only one patient from the SS-L patients in the first set of samples had previously received combination treatment with rituximab and chemotherapy.

### Serum Lp-PLA2 Activity by ELISA

The activity of Lp-PLA2 as measured by ELISA was also found to be significantly higher in SS-L compared to the SS-nL group (**Figure 1D**) (mean ± SD [nmol/min/ml]: 19.4 ± 4.5 *vs* 15.2 ± 3.3, p<0.0001). A similar difference was also observed when SS-L patients were compared to HC (mean ± SD [nmol/min/ml]: 19.4 ± 4.5 *vs* 14.5 ± 3.0, p-value: <0.0001). Lp-PLA2 activity did not differ between SS-nL patients and HC. After comparison of the three groups by ANOVA testing, the resulting p-value was less than 0.0001. When ROC curves were constructed to evaluate the distinguishing ability of Lp-PLA2 activity between SS-nL, SS-L, and HC, the corresponding AUC were 0.7696, CI (95%): 0.66–0.88, p-value: <0.0001 (**Figure 1E**) and 0.8114, CI (95%): 0.70–0.92, p-value: < 0.0001 for SS-L *vs* HC (**Figure 1F**). Among SS-nL patients of low or medium risk for lymphoma development no significant difference was detected (**Supplementary Figure 1B**). No statistically significant differences in Lp-PLA2 activity between SS-L patients previously treated with rituximab and/or chemotherapy compared to those with no previous treatment (**Supplementary Figure 2**).

### *PLA2G7* mRNA Expression in MSG Tissues

**Figure 2:**
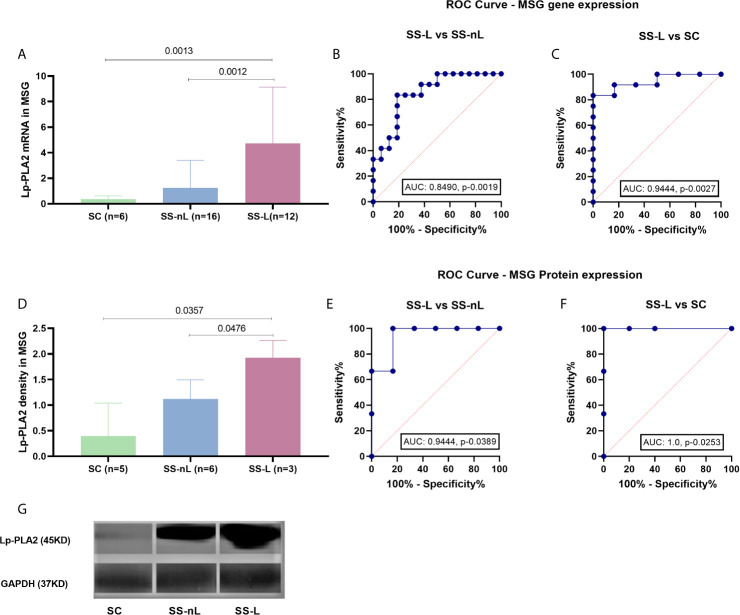
Lp-PLA2 (encoded by the PLA2G7 gene) mRNA expression (measured by real-time PCR) and protein density (tested by Western blot) in MSG tissues of our study participants and ROC curve analysis for both methods. **(A)** PLA2G7 gene expression was significantly increased in SS-L patients compared to both SS-nL patients and SC (p-values: 0.0012 and 0.0013, respectively). **(B)** ROC analysis could distinguish between SS-L and SS-nL patients with an AUC 0.8490, CI (95%): 0.71–0.99, p-value: 0.0019. **(C)** For SS-nL vs SC patients ROC curves showed an AUC 0.9444, CI (95%): 0.84–1.00, p- value: 0.0027. **(D)** Increased Lp-PLA2 protein expression was found in MSG total protein extracts derived from SS-L patients compared to both SS-nL patients and SC as tested by Western Blot (p-values: 0.0476 and 0.0357, respectively). **(E)** ROC analysis for Lp-PLA2 protein expression depicted an AUC of 0.94, CI (95%): 0.79–1.00, p-value: 0.0389 for SS-L vs SS-nL patients. **(F)** ROC analysis revealed an AUC of 1.00, CI (95%): 1.00–1.00, p-value: 0.0253. **(G)** Representative image of Lp-PLA2 and GAPDH MSG protein expression by Western Blot. SC, controls with sicca complaints; SS-n, Sjogren’s syndrome; SS-L, Sjogren’s syndrome complicated by lymphoma; ROC, receiver operating characteristic; AUC, area under the curve.

The mRNA expression of the *PLA2G7* gene was significantly increased in MSG tissues derived from SS-L compared to both SS-nL patients and SC (4.7 ± 4.4 *vs* 1.3 ± 2.1 *vs* 0.4 ± 0.3, p-values: 0.0012 and 0.0013, respectively). No significant difference was observed between SS-nL patients and sicca controls (p-value: 0.48) (**Figure 2A**). Following comparison of the three groups by ANOVA testing, the resulting p-value was 0.006. When ROC curves for the predictive models were fitted, AUC were 0.8490, CI (95%): 0.71–0.99, p-value: 0.0019 for SS-L *vs* SS-nL patients (**Figure 2B**) and 0.9444, CI (95%): 0.84–1.00, p-value: 0.0027 for SS-L *vs* SC (**Figure 2C**). A marginally significant association between focus score and Lp-PLA2 MSG gene expression was detected (r = 0.38, p-value: 0.06).

Concomitant LpLA2 salivary gland tissue gene expression and serum Lp-PLA2 activity was available for eight patients included in the study (four measured by liquid scintillation and four by ELISA). The corresponding Spearman’s correlation coefficients and p-values for the liquid scintillation and ELISA cohorts were r = 0.40, p-value = 0.75 and r = 0.80, p-value = 0.33.

### Lp-PLA2 Protein Expression in MSG Tissues by Western Blot

Increased Lp-PLA2 protein expression was observed in MSG total protein extracts derived from SS-L patients compared to both SS-nL patients and SC, as shown by Western blot analysis (1.93 ± 0.34 *vs* 1.12 ± 0.37 *vs* 0.39 ± 0.65, p-values 0.0476 and 0.0357, respectively, **Figure 2D**). No significant differences were detected between SS-nL and SC groups (SS-nL, 1.12 ± 0.37 *vs* SC, 0.39 ± 0.65, p-value: 0.08; **Figure 2D**). Following comparison of the three groups by ANOVA testing, the calculated p-value was 0.004. When ROC curves for the predictive models were fitted, AUC were 0.9444, CI (95%): 0.79–1.00, p-value: 0.0389 between SS-L *vs* SS-nL patients (**Figure 2E**) and 1.00, CI (95%): 1.00–1.00, p-value: 0.0253 for SS-L vs SC **(** **Figure 2F**). A representative image of Lp-PLA2 and GAPDH MSG protein expression by Western Blot is shown in **Figure 2G**. 

### Lp-PLA2 Protein Expression in MSG Tissues by Immunohistochemistry

**Figure 3:**
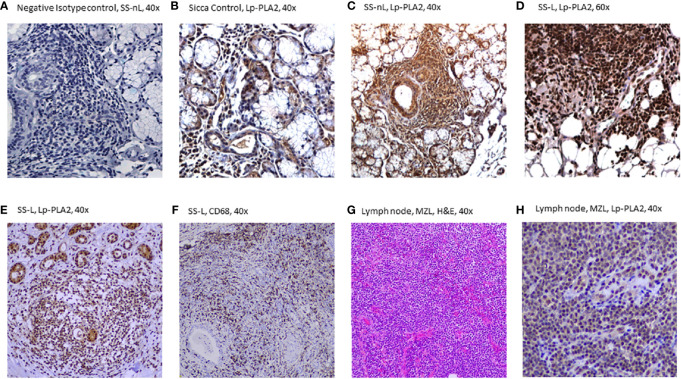
Lp-PLA-2 immunohistochemical expression in minor salivary gland (MSG) tissue sections from patients with sicca features (SC), Sjögren’s syndrome with no lymphoma (SS-nL), Sjogren’s syndrome with lymphoma (SS-L) **(A–F)**, as well as in a lymph node derived from a patient with marginal zone lymphoma **(G, H)**. **(A)** Isotype Control staining (grade 0), **(B)** Lp-PLA2 staining in SC (grade 1), **(C)** Lp-PLA2 staining in SS-nL (grade 2), **(D)** Lp-PLA2 staining in SS-L (grade 3), **(E)** Lp-PLA2 in SS-L, **(F)** CD68 staining (denoting the presence of tissue macrophages) in SS-L (same patient as **E**), **(G)** Hematoxylin & Eosin (H&E staining), marginal zone lymphoma (MZL), **(H)** Lp-PLA2 in a lymph node from a non-SS patient with MZL (same patient as **G**).

Increased Lp-PLA2 protein expression was found in MSG tissue sections derived from SS-L patients compared to both SS-nL patients and SC as tested by immunohistochemistry. **Figure 3A** displays a section of an MSG tissue derived from an SS-nL patient stained with isotype control. Lp-PLA2 protein expression was found mainly within lymphocytic infiltrates including tissue macrophages (the major Lp-PLA2–producing cell type) and lower expression was reported in salivary gland epithelial cells (**Figures 3B–F**). Of interest, immunohistochemical detection of Lp-PLA2 was detected in a lymph node derived from a non-SS patient with marginal zone lymphoma (**Figures 3G, H**).

### Lp-PLA2 Activity and Subclinical Atherosclerosis

Since the role of Lp-PLA2 activity in atherosclerosis is well established in the literature, we checked whether our results were influenced by the presence of atherosclerosis or risk factors for atherosclerosis in our groups. As shown in **Supplementary Table 1**, no significant differences in BMI, lipid, fibrinogen, or erythrocyte sedimentation rate (ESR) levels were detected between SS and SS-L groups. Additionally, no significant correlations were detected between serum Lp-PLA2 activity and systemic inflammation markers (fibrinogen, ESR) or lipid levels (**Supplementary Table 4**). While a borderline significant difference was detected in the frequency of elevated C-reactive protein (CRP) levels between SS-L and SS-nL patients (57.1 *vs* 18.4, p=value: 0.05) (**Supplementary Table 1**), no statistically significant difference in serum Lp-PLA2 activity levels was observed between patients with high *vs* normal CRP serum levels (57.9 ± 20.8 *vs* 47.7 ± 13.8, p=value: 0.20, data not shown). No significant differences were observed between SS patients (with or without the presence of lymphoma) and markers of subclinical atherosclerosis (plaque formation, presence of mean intima media thickness>0.9 in carotid and femoral arteries) (**Supplementary Figure 3**). No patient with lymphoma was on steroids at the time of blood sampling.

## Discussion

This is the first study to our knowledge in which a potential role of the Lp-PLA2 molecule in SS-related lymphomagenesis is revealed. After we applied two different methods, serum Lp-PLA2 activity was found to be significantly increased in SS patients complicated by lymphoma compared to both SS non lymphoma patients and healthy controls. In accord with the serum results, mRNA and protein expressions of the Lp-PLA2 molecule were also significantly increased in MSG tissue biopsy samples derived from SS patients with lymphoma compared to both SS patients with no lymphoma and sicca controls.

While liquid scintillation initially implemented in the first set of patients is considered the gold standard for Lp-PLA2 activity measurement by quantitation of [^3^H]PAF degradation products, there are certain limitations for widespread use in daily routine given the production of radioactive byproducts, the need for specific equipment and its time consuming nature. In this context, the extraction of similar results after application of the much easier and faster to perform ELISA method in an independent set of SS samples, holds promise for the use of Lp-PLA2 serum activity in clinical practice. Moreover, the high AUC values as determined by ROC analysis, as well the easy access to peripheral serum sample, imply Lp-PLA2 serum activity as a highly valuable serum predictive biomarker for lymphoma development in the context of SS.

While the mechanism through which deregulation of the Lp-PLA2 enzyme contributes to lymphoma development in the context of SS is not apparently clear, previous work supported a role for several phospholipases in tumorigenesis through generation of lipid mediators. The latter seem to get involved in cellular proliferation, migration, invasion and angiogenesis, previously designated as main pathogenic processes promoting malignant transformation (35).

Heightened Lp‐PLA2 expression has been found to be upregulated in several cancerous tissues including breast, colon, kidney, liver, or lung (36–38). Of note, in human and murine colon cancer cell lines, silencing of the gene expressing Lp-PLA2 (PLA2G7) led to reduction of tumor size by 42% *in vivo* and the PLA2G7 knockout Apc Min/+ mice displayed vigorous suppression of intestinal polyposis and colon tumor formation (37). In patients with prostate cancer, Lp‐PLA2 has been shown to promote cancer cell migration and invasion, possibly through generation of oxidized nonesterified fatty acids and lysophosphatidylcholine (LPC) (38). The latter is a phospholipid derivative following hydrolysis of oxidized phospholipids by Lp-PLA2 and has been previously shown to augment cell-to-cell adhesion molecules on endothelial cells, induce migration and proliferation of smooth muscle cells (39, 40) and along with oxidized non esterified fatty acids promote inflammatory effects (41). Collectively, these data reinforce the idea of the contribution of the inflammatory microenvironment in promoting malignant transformation.

Plasma Lp-PLA2 activity was previously measured in plasma derived from several autoimmune and inflammatory disorders. While modest increases have been earlier observed in patients with rheumatoid arthritis compared to controls with non-inflammatory arthritides (42), systemic sclerosis (43), or lupus anticoagulant-positive or aβ2GPI IgG positive sera (44), other studies in rheumatoid arthritis (45), systemic lupus erythematosus (23), juvenile rheumatoid arthritis (46), and Crohn’s disease (24), showed opposite results with a reduced Lp-PLA2 plasma activity being detected. Whether this is a primary defect or a result of increased disease activity leading to Lp-PLA2 inactivation through increased oxidation -known to occur in these disorders- remains to be elucidated (24). No data on SS have been so far available.

Given that Lp-PLA2 activity has been previously designated as a biomarker for atherosclerosis (47), we considered the possibility that different levels detected in Lp-PLA2 activity between SS-L and SS could be influenced by different levels of atherosclerotic burden or traditional risk factors for atherosclerosis. Since no associations between plaque formation, arterial wall thickening or cardiovascular contributors was detected, we assume that the increased Lp-PLA2 activity observed in SS patients with lymphoma are not influenced by underlying subclinical cardiovascular disease. Nevertheless, the relatively small number of sicca controls and the lack of available sera prior to lymphoma development are limitations of the current study.

Taken together, our results support a putative role of Lp-PLA2 serum activity, as well as salivary gland tissue Lp-PLA2 expression as novel contributors for lymphoma development in the setting of SS. The underlying molecular pathogenetic pathways leading to lymphomagenesis and the potential of serving as novel biomarker and therapeutic target remain to be explored in further longitudinal multicenter studies.

## Data Availability Statement

The original contributions presented in the study are included in the article/**Supplementary Material**. Further inquiries can be directed to the corresponding author.

## Ethics Statement

The studies involving human participants were reviewed and approved by the Laiko General Hospital of Athens and “G. Gennimatas” General Hospital of Athens Ethics Committee. The patients/participants provided their written informed consent to participate in this study.

## Author Contributions

EK, CM, and AN designed the study. All authors contributed to data collection. AN, CS, APs, KM, PG, AP, EK, and CM analyzed the data. AN, CS, PG, and EK critically interpreted the results and drafted the first version of the manuscript. All authors contributed to the article and approved the submitted version.

## Funding

Hellenic Rheumatology Society, Athens, Greece. Financial support by many donors to the Institute of Applied Physiology and Exercise in Medicine and HarmonicSS EU project 731944.

## Conflict of Interest

The authors declare that the research was conducted in the absence of any commercial or financial relationships that could be construed as a potential conflict of interest.
