# Supplementary material for: Lipoprotein-Associated Phospholipase A2: A Novel Contributor in Sjögren’s Syndrome-Related Lymphoma?
Source: Front Immunol. 2021 Jun 18;12:683623. doi: 10.3389/fimmu.2021.683623 (PMC8253309; doi:10.3389/fimmu.2021.683623)
Supplement: Supplementary file 1 [file DataSheet_1.docx]

**Suppl. Tables**

**Suppl. Table 1.** Demographic, clinical and laboratory features of SS patients according to lymphoma status (Radioimmunoassay cohort).

|  | **SS (n=50)** | **SS-lymphoma (n=30)** | **HC (n=32)** | **p-value** |
| --- | --- | --- | --- | --- |
| **Age (mean±S.D.)** | 59.7±10.5 | 61.8±9.6 | 55.5±9.0 | ns |
| **Female sex (%)** | 94.0 | 90.0 | 93.8 | ns |
| **Age of onset (mean±SD)** | 49.2±14.0 | 50.4±11.4 | na | ns |
| **Dry mouth (%)** | 89.6 | 96.7 | na | ns |
| **Dry eyes (%)** | 90.7 | 94.1 | na | ns |
| **Abnormal Schirmer's test (%)** | 88.5 | 84.0 | na | ns |
| **Rose Bengal Stain (≥4/9) (%)** | 30.8 | 50.0 | na | ns |
| **Antinuclear Antibodies ≥1/320 (%)** | 78.1 | 96.6 | na | ns |
| **Antibodies against Ro/SSA (%)** | 61.4 | 86.7 | na | ns |
| **Antibodies against La/SSB (%)** | 32.6 | 53.3 | na | ns |
| **Rheumatoid Factor positivity (>20 IU/ml)(%)** | 35.0 | 85.7 | na | ns |
| **Erythrocyte sedimentation rate (mm/h) (mean±SD)** | 24.0±19.4 | 38.6±30.6 | na | ns |
| **White Blood cell count (absolute number) (mean±SD)** | 5560 ±1725 | 5531±2239 | na | ns |
| **Neutrophil Number (absolute number) (mean±SD)** | 2971±1309 | 3548±1862 | na | ns |
| **Monocyte Number (absolute number) (mean±SD)** | 416±207 | 354.5±154 | na | ns |
| **Lymphocyte Number (absolute number) (mean±SD)** | 1768±677 | 1434±778 | na | ns |
| **Platelet Number (absolute number) (mean±SD)** | 226681±70500 | 246259±65136 | na | ns |
| **Low Complement 4 (<20mg/dl) (%)** | 40.0 | 73.3 | na | ns |
| **Hypergammaglobulinemia (>18%)** | 38.5 | 72.0 | na | ns |
| **Focus score (number of foci/4mm^2^) (mean±SD)** | 1.9±2.0 | 4.1±2.9 | na | ns |
| **Tarpley score (mean±SD)** | 1.9±1.0 | 3.3±0.6 | na | ns |
| **Salivary gland enlargement (%)** | 22.0 | 58.8 | na | ns |
| **Arthralgias/Myalgias (%)** | 63.6 | 56.7 | na | ns |
| **Arthritis (%)** | 20.5 | 13.3 | na | ns |
| **Raynaud's Phenomenon (%)** | 32.6 | 30.0 | na | ns |
| **Palpable Purpura (%)** | 9.3 | 23.3 | na | ns |
| **BMI (mean±SD)** | 26.4±5.7 | 27.2±8.7 | na | ns |
| **Cholesterol levels (mg/dl) (mean±SD)** | 191±39 | 204±31 | na | ns |
| **Triglyceride levels (mg/dl) (mean±SD)** | 107±45 | 96±24 | na | ns |
| **LDL (mg/dl) (mean±SD)** | 115±38 | 131±23 | na | ns |
| **HDL (mg/dl) (mean±SD)** | 56±16 | 54±11 | na | ns |
| **Fibrinogen (mg/dl) (mean±SD)** | 408±89 | 484±116 | na | ns |
| **ESR (mm) (mean±SD)** | 33.0±21.0 | 38.7±24.3 | na | ns |
| **High C-Reactive protein levels (%)** | 18.4 | 57.1 | na | 0.05 |

|  | **SS (n=50)** | **SS-lymphoma (n=9)** | **ΗC (n=42)** | **p-value** |
| --- | --- | --- | --- | --- |
| **Age (mean±S.D.)** | 58.2±13.0 | 63.4±11.6 | 55.0±10.2 | ns |
| **Female sex (%)** | 88.0 | 100.0 | 39 (92.9) | ns |
| **Age of onset (mean±SD)** | 47.9±15.4 | 52.2±10.7 | na | ns |
| **Dry mouth (%)** | 90.0 | 100.0 | na | ns |
| **Dry eyes (%)** | 90.0 | 100.0 | na | ns |
| **Abnormal Schirmer's test (%)** | 75.0 | 88.9 | na | ns |
| **Rose Bengal Stain (≥4/9) (%)** | 41.2 | 20.0 | na | ns |
| **Antinuclear Antibodies ≥1/320 (%)** | 89.6 | 100.0 | na | ns |
| **Antibodies against Ro/SSA (%)** | 70.8 | 100.0 | na | ns |
| **Antibodies against La/SSB (%)** | 45.8 | 55.6 | na | ns |
| **Rheumatoid Factor positivity (>20 IU/ml)(%)** | 38.3 | 88.9 | na | ns |
| **Erythrocyte sedimentation rate (mm/h) (mean±SD)** | 28.5±22.3 | 31.0±19.0 | na | ns |
| **Low Complement 4 (<20mg/dl) (%)** | 46.0 | 77.8 | na | ns |
| **White Blood cell count (absolute number)(mean±SD)** | 5877 ±2525 | 5567±1441 | na | ns |
| **Neutrophil Number (absolute number)(mean±SD)** | 3587±2248 | 3553±1255 | na | ns |
| **Monocyte Number (absolute number) (mean±SD)** | 395±195 | 475±175 | na | ns |
| **Lymphocyte Number (absolute number) (mean±SD)** | 1676±768 | 1362±569 | na | ns |
| **Platelet Number (absolute number) (mean±SD)** | 244028±74306 | 231556±54328 | na | ns |
| **Hypergammaglobulinemia (>18%)** | 48.9 | 44.4 | na | ns |
| **Focus score (number of foci/4mm^2^) (mean±SD)** | 1.8±1.1 | 3.2±2.9 | na | ns |
| **Tarpley score (mean±SD)** | 1.9±1.1 | 2.3±1.6 | na | ns |
| **Salivary gland enlargement (%)** | 15.0 | 66.7 | na | ns |
| **Arthralgias/Myalgias (%)** | 47.6 | 66.7 | na | ns |
| **Arthritis (%)** | 14.6 | 12.5 | na | ns |
| **Raynaud's Phenomenon (%)** | 14.3 | 55.9 | na | ns |
| **Palpable Purpura (%)** | 14.6 | 22.2 | na | ns |

**Suppl. Table 2**. Demographic, clinical and laboratory features of SS patients according to lymphoma status (ELISA cohort).

|  | **Radioimmunosssay cohort**  **(n=59)** | **ELISA cohort**  **(n=80)** |
| --- | --- | --- |
| **Age (mean±S.D.)** | 59.0±12.8 | 60.6±10.3 |
| **Female sex (%)** | 89.8 | 74(92.5) |
| **Age of onset (mean±SD)** | 48.7±14.6 | 49.7±12.9 |
| **Dry mouth (%)** | 91.5 | 72/78(92.3) |
| **Dry eyes (%)** | 91.5 | 67/73(91.8) |
| **Abnormal Schirmer's test (%)** | 77.4 | 44/51 (86.3) |
| **Rose Bengal Stain (≥4/9) (%)** | 38.5 | 13/31 (41.9) |
| **Antinuclear Antibodies ≥1/320 (%)** | 91.2 | 60/70(85.7) |
| **Antibodies against Ro/SSA (%)** | 75.4 | 53/74(71.6) |
| **Antibodies against La/SSB (%)** | 43.4 | 30/73(41.1) |
| **Rheumatoid Factor positivity (>20 IU/ml) (%)** | 46.4 | 38/68(55.9) |
| **Erythrocyte sedimentation rate (mm/h) (mean±SD)** | 29.2±20.1 | 29.7±22.2 |
| **Low Complement 4 (<20mg/dl) (%)** | 50.8 | 42(52.5) |
| **White Blood cell count (absolute number) (mean±SD)** | 5818±2346 | 5548±1930 |
| **Neutrophil Number (absolute number) (mean±SD)** | 3580±2083 | 3198±1564 |
| **Monocyte Number (absolute number) (mean±SD)** | 410±192 | 395±192 |
| **Lymphocyte Number (absolute number) (mean±SD)** | 1616±732 | 1637±732 |
| **Platelet Number (absolute number) (mean±SD)** | 241533±70385 | 235492±68278 |
| **Hypergammaglobulinemia (>18%)** | 48.2 | 33/64 (51.6) |
| **Focus score (number of foci/4mm^2^) (mean±SD)** | 2.4±2.3 | 2.7±2.6 |
| **Tarpley score (mean±SD)** | 1.9±1.2 | 2.5±1.1 |
| **Salivary gland enlargement (%)** | 24.5 | 27/70(38.6) |
| **Arthralgias/Myalgias (%)** | 51.0 | 45/74(60.8) |
| **Arthritis (%)** | 14.3 | 13/74(17.6) |
| **Raynaud's Phenomenon (%)** | 21.6 | 23/73(31.5) |
| **Palpable Purpura (%)** | 15.8 | 11/73(15.1) |

**Suppl. Table 3**. Clinical characteristics in the two SS cohorts. No statistically significant differences were reported between SS cohorts.

**Suppl. Table 4.** Non statistically significant correlations between Lp-PLA2 activity with Body Mass Index (BMI), serum inflammatory markers and lipid levels.

|  | **rho**  **(spearman’s correlation coefficient)** | **p-value** |
| --- | --- | --- |
| **BMI** | -0.06 | 0.75 |
| **ESR (mm/h)** | 0.20 | 0.20 |
| **Fibrinogen (mg/dl)** | -0.03 | 0.20 |
| **Cholesterol levels (mg/dl)** | -0.21 | 0.21 |
| **Triglyceride levels (mg/dl)** | 0.09 | 0.59 |
| **LDL levels (mg/dl)** | -0.18 | 0.30 |
| **HDL levels (mg/dl)** | -0.23 | 0.17 |

**Suppl. Figures**

**Suppl Fig. 1.** Lp-PLA2 activity in patients with Sjogren’s syndrome (SS) according to the number of risk factors previously shown to predict lymphoma development. As shown in panels A and B, no significant differences were found between SS-no lymphoma patients with ≤ 2 adverse risk factors (low risk) compared to those presenting with 2-6 risk factors (medium risk). Patients with all 7 high risk factors (high risk) were not present in the two cohorts included in the present study.

**Suppl. Fig. 2.** No statistically significant differences in Lp-PLA2 activity were detected between SS-lymphoma patients previously treated with rituximab and/or chemotherapy compared to those with no previous treatment.

**Suppl Fig.3.** Lp-PLA2 activity in patients with Sjogren’s syndrome (SS) with and without the presence of atherosclerotic plaque formation and intima media thickness>0.9. No difference was found between SS patients without plaque and with the presence of plaque [mean±SD (nmol/min/ml): 49.2±16.4 vs 49.8±16.7, p-value: 0.68] and between patients with IMT<0.9 and patients with IMT>0.9 [mean±SD (nmol/min/ml): 46.1±10.2 vs 49.5±15.0, p-value: 0.50].
